# Risk assessment of mouse gastric tissue cancer induced by dichlorvos and dimethoate

**DOI:** 10.3892/ol.2013.1155

**Published:** 2013-01-28

**Authors:** QING-LU WANG, YU-JUN ZHANG, CAI-XIA ZHOU, JIE ZHANG, YE DOU, QIAO-QIAO LI

**Affiliations:** Key Laboratory of Biomedical Engineering and Technology of Shandong High School, Shandong Wanjie Medical College, Zibo 255213, P.R. China

**Keywords:** gastric cancer, dichlorvos, dimethoate, transcription, mouse

## Abstract

Cancer hazards from pesticide residues in food have been much discussed in the past decade. In this study, we showed that dichlorvos and dimethoate affect hemoglobin content and hematocrit value, but had no effect on red blood cell counts and total plasma protein in mice. A 40-mg/kg/day dose of dichlorvos upregulated the expression of *p16*, *Bcl-2* and *c-myc* genes in mouse gastric tissue. By contrast, expression of the *p16*, *Bcl-2* and *c-myc* genes induced by low doses (5, 10 and 20 mg/kg/day) of dichlorvos demonstrated no change in the control check group (CK; 200 *μ*l sterile saline perfused group; 0 mg/kg/day). Different doses of dimethoate all upregulated the expression of *p16*, *Bcl-2* and *c-myc* genes in mouse gastric tissue. The results further demonstrated that mouse gastric tissue, exposed in the long-term to low doses of dichlorvos and dimethoate, has the potential to become cancerous.

## Introduction

It has been observed that gastric cancer incidence is elevated in farm workers involved in the cultivation of citrus fruits and in workers exposed to high levels of the herbicides 2,4-D and trifluran, the insecticides chlordane and malathion, the fungicides mancozeb and maneb, the fumigant methyl bromide and the acaricide propargite (1). Four of the chemicals associated with elevated gastric cancer incidence (chlordane, maneb, mancozeb and propargite) are class B2 chemicals (probable carcinogens) as classified by the United States Environmental Protection Agency (2006), while trifluran and simazine are class C chemicals (possible carcinogens).

The gastric cancer subsite distribution was approximately the same between farm workers and Hispanic males in California from 1990–1994. Among the farm workers, 14% of cancer cases were of the gastric cardia compared with 18.7% in Californian Hispanic males (2). Similarly, among the farm workers, 61% of cancer cases were classified as adenocarcinoma, not otherwise specified, and 64% of Californian Hispanic males were also of this histological type (1).

Dichlorvos has been widely used as an insecticide for >50 years. Its carcinogenic potential has been studied extensively and in total there have been 11 long-term studies in rodents; six in rats and five in mice (3). Numerous studies have been carried out on the toxicity of dimethoate in non-target animals and humans. Dimethoate was demonstrated to have an effect on reproductive and endocrine function, enzymatic changes, immunotoxicity, brain acetylcholinesterase activity, proteins and carbohydrate metabolism (4–8).

Few studies have been carried out on the effects of dimethoate in gastric cancer. The present study aimed to investigate the changes in *p16*, *Bcl-2* and *c-myc* gene expression in the gastric tissue of mice following perfusion with the organophosphorous pesticides dichlorvos and dimethoate, which are extensively used in Chinese agricultural areas.

## Materials and methods

### Animals and treatment

Male Kunming mice, weighing 180–200 g, were divided randomly into five equal groups, each containing 6 male mice. The mice were purchased from Shandong University (Shandong, China). The mice were routinely screened for common mouse pathogens. The mice used in this study were housed in groups of 6 in stainless steel cages (50×40×25 cm) in rooms determined to be free of common pathogens under standard conditions (24±2°C and 50±5% humidity) with a 12-h light-dark cycle. The groups were perfused with 0, 5, 10, 20 or 40 mg/kg/day dichlorvos suspended in sterile saline (200 *μ*l, 0.9% NaCl) or 0, 2.5, 5, 10 or 20 mg/kg/day dimethoate suspended in sterile saline (200 *μ*l, 0.9% NaCl). With the exception of during treatment, the mice had free access to food and water.

After three weeks, all mice were killed by decapitation for the experiments. The esophagus and duodenum were removed and gastric tissue was excised and washed with sterile saline. The tissue was rapidly frozen in liquid nitrogen and stored at −80°C. All animal procedures were approved by Shandong Wanjie College Animal Investigational Committee and performed in accordance with the Guide for the Care and Use of Laboratory Animals published by Ministry of Health People’s Republic of China.

### Hematological investigation

Blood samples were collected from the hearts of the mice for hematological investigation. The parameters investigated were the hemoglobin content, hematocrit value (PCV), red blood cell count and total plasma protein (9–11).

### Expression analysis of p16, Bcl-2 and c-myc by real-time quantitative (qRT)-PCR

Total RNA was isolated from <100 mg of tissue by using TRIzol Reagent (Invitrogen Life Technologies, Carlsbad, CA, USA) according to the manufacturer’s instructions. Total RNA was quantified by determination of the optical density at 260 nm. First-strand cDNA was produced following instructions for AMV reverse transcriptase (AMV RT) with slight modifications. In the reverse transcription reaction system, 2 *μ*g total RNA, 10 *μ*l AMV buffer, 75 pmol oligo(dT_18_) and 5 *μ*l of each of the four deoxynucleotide triphosphates (10 mM) were contained in a total 50 *μ*l reaction volume. The mixture was incubated at 95°C for 5 min and then 40 units RNase, 10 units AMV RT (Promega, Madison, WI, USA) and 7.5 *μ*l 25 mM MgCl_2_ were added. The mixture was incubated at 42°C for 60 min and heated to 95°C for 5 min to inactivate the reverse transcriptase. The cDNA product was stored at −20°C.

Oligonucleotides for qRT-PCR (Table I) were designed using Primer Premier 5.0 software (Premier Biosoft International). A BLAST analysis was used against other organism genome sequences for specificity confidence (http://www.ncbi.nlm.nih.gov/BLAST/). The Mfold web server was applied to avoid positioning on unstable secondary structures. The primer specificity was analyzed before qRT-PCR.

The reaction mixture (final volume, 20 *μ*l) consisted of 10 *μ*l SYBR *Ex Taq* II (Applied Biosystems, Carlsbad, CA, USA), 0.4 *μ*l ROX reference dye, 0.8 *μ*l of each primer (final concentration*,* 250 nM), 7 *μ*l ddH_2_O and 1 *μ*l cDNA (dilution factor, 1/10). The thermocycling program consisted of two phases; one cycle at 95°C for 4 min, 40 cycles at 95°C for 15 sec, 52°C for 25 sec and 72°C for 35 sec. Following completion of these cycles, melting-curve data were collected to verify the PCR specificity, contamination and absence of primer dimers. Each sample was tested in triplicate in a StepOnePlus™ Real-Time PCR apparatus (Applied Biosystems).

The relative expression levels of *p16, Bcl-2* and *c-myc* were normalized against GAPDH. The normalized relative gene expression levels (indicated as n-fold induction) were evaluated by the Real-Time PCR System Cycler software. The formula was determined as: ΔCt_treatment_ (the threshold cycle of MT of treated *T. thermophila*) = Ct_MT treatment_ − Ct_17S treatment_; ΔCt_control_ (the threshold cycle of MT of controlled *T. thermophila*) = Ct_MT control_ − Ct_17S control_; ΔΔCt = Ct_treatment_ − Ct_control_; and the relative quantity levels of MT mRNA expression were 2^−CtΔΔ^ (12).

### Statistical analysis

All hematological results are presented as the mean ± SD. Data were evaluated using SPSS software (SPSS company, USA). Statistical differences were determined using Student’s t-test with a significance level set at P<0.05.

## Results

### Hematological studies

At the end of the treatment period, no teratogenic mice were observed. The blood parameters investigated were the hemoglobin content, PCV, red blood cell counts and total plasma protein. The results were statistically analyzed using Student’s t-test and are shown in Table II.

**Figure 1:**
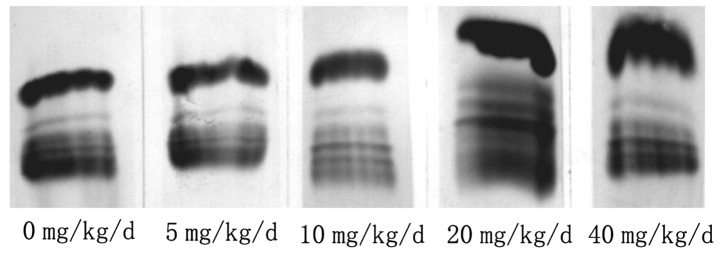
Effects of dichlorvos on the bands of plasma proteins. The electrophoretogram of plasma proteins from mice perfused with 0, 5, 10, 20 or 40 mg/kg/day dichlorvos suspended in sterile saline (200 *μ*l, 0.9% NaCl) for three weeks were analyzed using cellulose acetate membrance electrophoresis. The fractions that appeared clearly were albumin and α-, β- and γ-globulins and these fraction ratios (gray ratio) did not change significantly.

The hemoglobin content and PCVs showed significant decline in mice treated with 20 mg/kg/day dichlorvos (P<0.05) when compared with control values and the decline was also significant in mice treated with 40 mg/kg/day dichlorvos (P<0.001). The plasma protein was investigated quantitatively and qualitatively. The total plasma protein content was not significantly different between the mice treated with different doses of dichlorvos and those from the control group. The electrophoretic investigation showed no significant differences between the differential bands of plasma proteins in the treated and control mice (Fig. 1). The fractions that appeared clearly were albumin and α-, β- and γ-globulins. The red blood cell counts from control and treated mice are shown in Table III. The red blood cell counts in mice treated with each of the dichlorvos concentrations were not significantly different from those of the controls (P>0.05).

**Figure 2:**
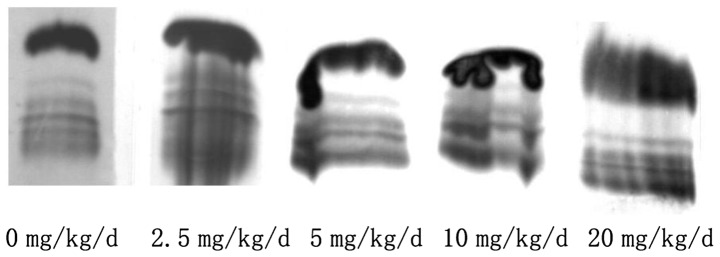
Effects of dimethoate on the bands of plasma proteins. The electrophoretogram of plasma proteins from mice perfused with 0, 0.5, 5, 10 or 20 mg/kg/day dimethoate suspended in sterile saline (200 *μ*l, 0.9% NaCl) for three weeks were analyzed using cellulose acetate membrance electrophoresis. The fractions that appeared clearly were albumin and α-, β- and γ-globulins and these fraction ratios (gray ratio) did not change significantly.

Compared with the control mice, mice treated with 5, 10 or 20 mg/kg/day dimethoate showed a change in their hemoglobin content and PCVs. There was a significant decline in the 5mg/kg/day group, followed by the 10 mg/kg/day group and in the 20 mg/kg/day group, the decline was the most evident (Table III). The albumin and α-, β- and γ-globulins appeared clearly in the electrophoretic fractions for the total plasma protein and their concentration ratios did not change significantly (Fig. 2). The red blood cell counts in mice treated with each of the dimethoate concentrations were not significantly different from those of the controls (P>0.05).

### Effects of dichlorvos on expression of the p16, Bcl-2 and c-myc genes

**Figure 3:**
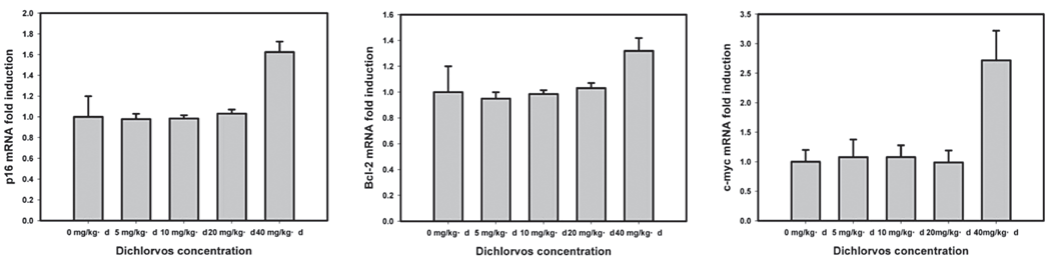
Effects of dichlorvos on the expression levels of the *p16*, *Bcl-2* and *c-myc* genes. Relative expression levels of the *p16*, *Bcl-2* and *c-myc* genes were obtained by real-time quantitative PCR. Fold-induction was determined for the *p16*, *Bcl-2* and *c-myc* genes after mice were perfused with 0, 5, 10, 20 or 40 mg/kg/day dichlorvos suspended in sterile saline (200 *μ*l, 0.9% NaCl) for three weeks. Gene expression levels are shown in comparison with an untreated control.

Comparative gene expression analysis of the *p16*, *Bcl-2* and *c-myc* genes was carried out under various concentration stress conditions using qRT-PCR. Results show mRNA expression levels of the *p16*, *Bcl-2* and *c-myc* genes induced by various concentration stressors, normalized against the level of GAPDH (the reference gene). In dichlorvos induction, the *p16*, *Bcl-2* and *c-myc* expression levels showed no clear change with low dichlorvos concentrations (<20 mg/kg/day), however, levels subsequently increased with 20- to 40-mg/kg/day dichlorvos concentrations. Fig. 3 shows that the effect of dichlorvos on *Bcl-2* (1.3-fold) was the lowest compared with the upregulated expression fold, the next was *p16* (1.7-fold) and *c-myc* (2.8-fold) demonstrated the greatest effects.

### Effects of dimethoate on expression of the p16, Bcl-2 and c-myc genes

**Figure 4:**
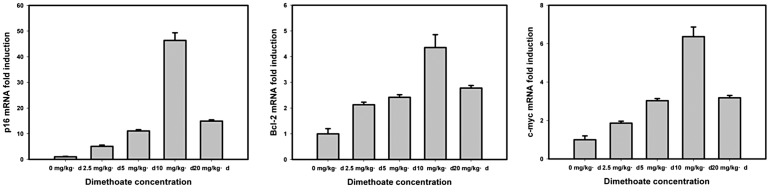
Effects of dimethoate on the expression levels of the *p16*, *Bcl-2* and *c-myc* genes. Relative expression levels of the *p16*, *Bcl-2* and *c-myc* gene were obtained by real-time quantitative PCR. Fold-induction was determined for the *p16*, *Bcl-2* and *c-myc* genes after mice were perfused with 0, 2.5, 5, 10 or 20 mg/kg/day dimethoate suspended in sterile saline (200 *μ*l, 0.9% NaCl) for three weeks. Gene expression levels are shown in comparison with an untreated control.

Expression of the *p16*, *Bcl-2* and *c-myc* gene transcripts was assessed after *in vivo* exposure to dimethoate. The concentrations used were <1/2 the LC50 value of the mouse. In dimethoate induction, the *p16*, *Bcl-2* and *c-myc* expression levels gradually increased with concentrations <10 mg/kg/day and subsequently decreased with 10- and 20-mg/kg/day dimethoate concentrations (Fig. 4). The effect of dimethoate on the expression of *Bcl-2* (4.5-fold) was the lowest, the next was *c-myc* (6.1-fold) and *p16* (48-fold) demonstrated the greatest effects.

## Discussion

In the past decade, the incidence of gastric cancer has been declining, however it remains the fourth most common cancer and the second most frequent cause of cancer-associated mortalities (13,14). The reason for the decline in gastric cancer incidence is possibly due to the recognition of specific risk factors, including *H. pylori*, and dietary and environmental risks. However, gastric cancer is the predominant type of cancer in farmers in the developing world, particularly China, and remains a significant cancer burden (15).

In this study, the risks to mouse gastric tissue were analyzed by continuous exposure to low doses of dichlorvos and dimethoate. We showed that red blood cell counts and total plasma protein in these mice were not affected by dichlorvos and dimethoate, while hemoglobin content and PCVs demonstrated a significant decline in treated mice when exposed to higher doses of dichlorvos (20–40 mg/kg/day) and dimethoate (5–20 mg/kg/day). These results suggest that the pesticides in this study were able to affect the physiological functions of mice, however, the impacts were small.

A number of biological markers, including oncogenes, tumor suppressor genes, cell cycle regulators and DNA repair genes, were correlated with the genesis, growth, invasion and metastasis of tumors and they may be used as prognostic factors for tumors (16). Currently, numerous studies have evaluated the prognostic significance of markers, such as the *p16*, *Bcl-2*, *MTDH* and *c-myc* genes in gastric cancer (17–19). This study also evaluated expression of the *p16*, *Bcl-2* and *c-myc* genes. Results showed that expression of the *p16*, *Bcl-2* and *c-myc* genes in mouse gastric tissue was not affected when it was exposed to low concentrations of dichlorvos (5, 10 or 20 mg/kg/day), however, there was a significant increase in expression levels with a 40-mg/kg/day dose of dichlorvos. These results suggest the risk of tumorigenesis caused by dichlorvos was substantial, but not high, when mice were continuously exposed to dichlorvos. The risk of tumorigenesis induced by dimethoate was higher than for dichlorvos. Low doses of dimethoate were able to induce expression of the *p16*, *Bcl-2* and *c-myc* genes in mouse gastric tissue. There was a positive correlation between dimethoate concentration and expression levels of the *p16*, *Bcl-2* and *c-myc* genes. In the 20 mg/kg/day group, the cause of the apparent decline in expression levels of these genes may be due to the function of cells being affected.

In conclusion, cancer hazards from pesticide residues in food have been much discussed in decades past. Our results suggest that mouse gastric tissue exposed long-term to low dose dichlorvos and dimethoate has the potential to become cancerous. However, the mechanisms of gastric tissue cancerization induced by pesticide remains to be elucidated by further studies in the future.

## Figures and Tables

**Table I:** Real-time quantitative PCR primers.

Primer	Sequence (5′-3′)
GAPDHF	TGTGTCCGTCGTGGATCTGA
GAPDHR	CCTGCTTCACCACCTTCTTGA
p16F	GTACCCCGATTCAGGTGATG
p16R	TTAGCTCTGCTCTTGGGATTG
Bcl-2F	AGGAGCAGGTGCCTACAAGA
Bcl-2R	GCATTTTCCCACCACTGTCT
c-mycF	GGTGGAAAACCAGGTAAGCA
c-mycR	CCTTCTCCTCTGCCATCTTC

**Table II:** Hematological studies of mice exposed to dichlorvos.

	Dichlorvos dose (mg/kg/day)				
Parameter	0	5	10	20	40
Hb (mg/100 ml)	13.33±1.97	12.83±2.12	12.15±2.13	10.95±1.59^a^	9.85±1.67^b^
PCV (%)	41.0±2.77	40.35±3.17	39.86±4.69	34.9±3.68^a^	32.63±4.41^b^
Total plasma protein (×10^12^/l)	50.52±6.22	50.64±6.20	50.0±5.98	50.02±6.08	49.08±7.21
RBC count (mg/100 ml)	7.41±0.32	7.37±0.40	7.42±0.22	7.38±0.96	7.39±0.26

Results are presented as mean ± SD.

a P<0.05, 0 mg/kg/day dose group vs. 5, 10, 20 or 40 mg/kg/day dose groups.

b P<0.001. Hb, hemoglobin; PCV, hematocrit value; RBC, red blood cell.

**Table III:** Hematological studies of mice exposed to dimethoate.

	Dimethoate dose (mg/kg/day)				
Parameter	0	2.5	5	10	20
Hb (mg/100 ml)	13.33±1.97	12.93±2.13	11.17±1.11^a^	9.55±1.59^a^	8.05±1.67^b^
PCV (%)	41.0±2.77	40.45±3.14	37.86±1.08^a^	36.4±1.68^a^	33.67±0.71^b^
Total plasma protein (mg/100 ml)	50.52±6.22	50.65±6.01	50.32±5.78	49.02±6.38	49.08±7.67
RBC count (×10^12^/l)	7.41±0.32	7.40±0.29	7.38±0.82	7.39±0.86	7.37±0.76

Results are presented as mean ± SD.

a P<0.05, 0 mg/kg/day dose group vs. 2.5, 5, 10 or 20 mg/kg/day dose groups.

b P<0.001. Hb, hemoglobin; PCV, hematocrit value; RBC, red blood cell.
